# Complete genome analysis and characterization of neurotropic dengue virus 2 cosmopolitan genotype isolated from the cerebrospinal fluid of encephalitis patients

**DOI:** 10.1371/journal.pone.0234508

**Published:** 2020-06-18

**Authors:** Mya Myat Ngwe Tun, Rohitha Muthugala, Takeshi Nabeshima, Aung Min Soe, Shyam Prakash Dumre, Lakmali Rajamanthri, Dulani Jayawardana, Shanthi Attanayake, Shingo Inoue, Kouichi Morita

**Affiliations:** 1 Department of Virology, Institute of Tropical Medicine, Nagasaki University, Nagasaki, Japan; 2 Department of Virology, Teaching Hospital Kandy, Kandy, Sri Lanka; 3 Department of Immunogenetics, Institute of Tropical Medicine, Nagasaki University, Nagasaki, Japan; Defense Threat Reduction Agency, UNITED STATES

## Abstract

Dengue virus (DENV) infection remains a major public health concern in many parts of the world, including Southeast Asia and the Americas. Sri Lanka experienced its largest dengue outbreak in 2017. Neurological symptoms associated with DENV infection have increasingly been reported in both children and adults. Here, we characterize DENV type 2 (DENV-2) strains, which were isolated from cerebrospinal fluid (CSF) and/or serum of patients with dengue encephalitis. Acute serum and CSF samples from each patient were subjected to dengue-specific non-structural protein 1 (NS1) antigen test, IgM and IgG enzyme-linked immunosorbent assay (ELISA), virus isolation, conventional and real-time polymerase chain reaction (PCR), and next-generation sequencing (NGS). Among the 5 dengue encephalitis patients examined, 4 recovered and 1 died. DENV-2 strains were isolated from serum and/or CSF samples of 3 patients. The highest viral genome levels were detected in the CSF and serum of the patient who succumbed to the illness. A phylogenetic tree revealed that the DENV-2 isolates belonged to a new clade of cosmopolitan genotype and were genetically close to strains identified in China, South Korea, Singapore, Malaysia, Thailand, and the Philippines. According to the NGS analysis, greater frequencies of nonsynonymous and synonymous mutations per gene were identified in the nonstructural genes. The full genomes of serum- and CSF-derived DENV-2 from the same patient shared 99.7% similarity, indicating that the virus spread across the blood-brain barrier. This is the first report to describe neurotropic DENV-2 using whole-genome analysis and to provide the clinical, immunological, and virological characteristics of dengue encephalitis patients during a severe dengue outbreak in Sri Lanka in 2017.

## Introduction

Dengue is one of the most globally prevalent, arthropod-borne, viral diseases in humans [1]. The overall incidence of dengue, as well as the incidence of explosive dengue outbreaks, has increased dramatically over the last several years [2]. The causative agent, dengue virus (DENV), which includes four distinct, but closely related serotypes, belongs to the genus *Flavivirus* in the family *Flaviviridae* [3]. Transmitted by *Aedes* mosquitoes, dengue virus occurs primarily in tropical and subtropical areas of the world [4]. The infection causes a flu-like illness, and patients occasionally develop potentially lethal complications. The different degrees of dengue severity were re-categorized in 2009 by the World Health Organization (WHO) into dengue without warning signs (DwoWS), dengue with warning signs (DwWS), and severe dengue (SD) [5]. The annual incidence of dengue infections was estimated to be 400 million per year, of which approximately 96 million were clinically apparent [2]. Death occurs in about 2.5% of dengue-infected people [2, 3]. In recent years, there has been an increase in the number of reported cases of neurological manifestations associated with dengue infections. However, the precise incidence rate of neurological symptoms remains unclear [6]. Neurological signs were first reported in 1976 as atypical symptoms of dengue infection, and their incidence rates have varied from 0.5% to 20% in recent years [7, 8]. Neurological complications associated with DENV infection include encephalopathy (caused by hepatic failure or metabolic disorders), encephalitis (caused by direct viral invasion), neuromuscular complications (Guillain-Barre syndrome or transient muscle dysfunctions), and neuro-ophthalmic involvement [9]. In addition, other less common neurological features have been described as atypical manifestation of dengue infection. Dengue serotypes 2 and 3 are most commonly associated with neurological symptoms [10, 11]. Confirmed dengue cases with neurological manifestations have been confirmed by assessing the presence of the virus and/or antibody in the cerebrospinal fluid (CSF) [6, 12]. However, molecular and biological characterizations of neurotropic DENV strains have been extremely limited, despite their important roles in the neuropathogenesis of dengue.

In 2017, the largest dengue outbreak was reported in Sri Lanka, with over 185,000 clinical cases and at least 250 fatal cases [13]. The age distribution of infected individuals showed that many patients were young people (15–39 years age group) [13]. Atypical manifestations of DENV infection, i.e. dengue encephalitis, were reported during this outbreak. The aims of our study were to describe the neurotropic DENV-2 strains that we isolated from CSF and serum samples of pediatric and adult patients with dengue encephalitis during the severe dengue outbreak in Sri Lanka, in 2017 and to provide clinical, immunological, and virological characteristics of these patients.

## Materials and methods

### Ethics statement

Ethical approvals for this study were provided by the Institution Ethical Committee on Medical Research and Review, General Hospital (Teaching) Kandy, Sri Lanka (THK/ERC/73/2017) and the Institute of Tropical Medicine Ethical Committee, Nagasaki University, Japan (180608200).

### Sample collection

Paired serum and CSF samples used in this study were from five confirmed DEN patients between March 2017- January 2018. These patients included children (< 15 years old) and adults admitted to Teaching Hospital Kandy, Sri Lanka. Confirmation of dengue infection was based on the clinical findings supported by laboratory tests. Patients were classified as having DwoWS or DwWS or SD according to WHO 2009 guideline [5]. Informed Consent was obtained from patients or parents of children or their legal guardians prior to the collection of samples.

### DENV IgM and IgG ELISA tests

Serological confirmation of DENV infection was done by an in-house DENV IgM capture ELISA and anti-DENV IgG indirect ELISA. NS1 antigen from serum samples was detected by using SD BIOLINE Dengue NS1 antigen (Ag) rapid test (Standard Diagnostic Inc., Korea). To confirm DENV infection, the in-house DENV IgM was performed following the procedure described previously [14, 15]. Optical density (OD) was read at 492nm and P/N (positive control or sample OD/ negative control OD) ratio ≥ 2 was considered as positive. To determine primary and secondary DENV infections, we used our in-house DENV IgG indirect ELISA [16] which had a high correlation with dengue hemagglutination inhibition (DEN HI) test, the gold standard recommended by the World Health Organization [17]. If the IgG titer was ≥ 29,000, infection was considered secondary, whereas a titer < 29,000 was considered primary [16].

### Virus isolation in cell culture

To isolate DENV, serum and CSF samples at 10 ul volume each were inoculated onto *Aedes albopictus* clone mosquito cells (C6/36 E2) grown in flat culture tubes. Infected cells were incubated at 28°C for 7 days in Eagle’s Minimum Essential Medium supplemented with 2% fetal calf serum and 0.2 mM of non-essential amino acids [18]. The infected culture fluid (ICF) from each tube was collected, aliquoted and stored at -80**°**C until use. A second virus passage was done in tubes with fresh confluent cells which were incubated for one week with the same incubation conditions as the first passage. ICF from each tube was collected and processed as before.

### RNA extraction and conventional RT-PCR

To test ICFs for the presence of DENV, RNA was extracted from them by using viral RNA mini kit (Quiagen, Hilden, Germany) according to the manufacturer’s instruction. Then, PrimeScript^™^ One Step RT-PCR Kit Ver.2 (Takara Bio Inc., Shiga, Japan) was used following the manufacturer’s instruction. A volume of 5 μl of RNA was used for conventional RT-PCR and amplification was done by using a total of 25 μl of reaction mixture consisting of 1 μl of enzyme mix, 13 μl of 2x buffer, 4 μl of nuclease water, 1 μl of 100 pmol forward and reverse primers with separate primer sets for the detection of DENV and determination of specific DENV serotypes [19–21].

### Quantification of DENV genome levels

Viral RNA was directly extracted from 140 μl of patient serum and the same kit was used to extract RNA from ICF. A volume of 5 μl of RNA was used for quantitative real time RT-PCR (qRT-PCR) and amplification of the envelope gene was performed by using a total of 20 μl of reaction mixture consisting of 5 μl of Taqman master mix, 9 μl of nuclease water, 0.3 μl of 100 pmol forward and reverse primers, 0.4 μl of probe with DENV serotype specific primers of TaqMan Fast Virus 1-Step Master Mix (Life Technologies, CA,USA), following the protocol described in a previous report [22, 23]. The viral genome levels were expressed as log_10_ genome copies/ml.

### Full length viral genome amplification and phylogenetic analysis

Whole transcriptome libraries (Ion Total RNA-Seq Kit v2, Life Technologies, CA, USA) were synthesized by using RNA extracted from ICF. Sequencing was conducted by Next-Generation Sequencing (NGS), Ion Proton (Life Technologies, CA, USA). The low quality reads that had < 75% with quality score of < 20 were removed by FASTX-Toolkit Version (v) 0.0.14 from the input data file. Before and after the quality trimming, sequence quality was checked by FastQC v 0.11.8. For the de novo assembly, Trinity v 2.8.4 [24] was used and the sequence name was repaired by Seqkit v 10.0.1. while blastn v 2.7.1 [25] was used to the assembled de novo contig. Trimmed fastq data set were mapped by bwa v 0.7.17 [26] to the reference sequence chosen by blastn, and variants were detected by LoFreq v 2.1 3.1 [27], and Varscan v 2.4.3 [28]. From the output of Varscan, Samtools v 1.9 constructed the consensus dengue virus sequence. Data pre-processing was conducted according to the best practice workflow for GATK v 3.8.1 and Picard v 2.20. Dengue virus sequences in the International Nucleotide Sequence Database Consortium were collected and annotated with Entrez-edirect and annotated by Seqkit. The sequences of the full genome coding region were aligned by Mafft v 7.407 [29]. Maximum likelihood phylogenetic trees were constructed by Phyml v 3.2.0 [30]. Bootstrap values were obtained after 1000 replications. The substitution model was selected by jModelTest v 2.1.10 [31].

### Statistical analysis

Data were analyzed by using SPSS for Windows, version 22.0 (IBM Corp., Armonk, NY). Categorical variables were presented as absolute number (n) and percentage (%) as appropriate. Chi-square test (or Fisher’s exact test) was used to determine association among the categorical variables. Comparison of continuous variables (ratio of non-synonymous vs synonymous mutations) was done by Mann-Whitney U test. An alpha level of 0.05 was used for all statistical analyses and a *P*-value less than 0.05 was considered statistically significant.

## Results

### Characteristics of patients with neurological manifestations

During the 2017 severe dengue outbreak in Sri Lanka, 295 patients suspected with dengue infection were admitted to the Kandy Teaching Hospital in Kandy, Sri Lanka. There were five patients who showed neurological manifestations. Paired serum and CSF samples were collected from these patients (2 children and 3 adults) who were diagnosed with SD (Table 1). All patients experienced the following symptoms: fever; joint, muscle and eye-socket pain; nausea; vomiting; headache; irritability and; photophobia. Of the 5 patients, 4 recovered and 1 died. The patient who died was a 19-year-old female. She had abdominal pain, liver enlargement, free fluid in the abdomen/pelvis, confusion, loss of consciousness, and generalized fits. She required ventilation. The 4 patients who recovered had signs and symptoms of SD, including neck stiffness, alteration of mental status, confusion, and liver enlargement. The CSF analysis of the samples from all 5 patients revealed low levels of white blood cells (WBCs) and protein, high glucose levels, and a high percentage of CSF/blood sugar, whereas, the serum samples showed low WBC counts and platelet levels (Table 1), leading to the diagnosis of leukopenia-associated thrombocytopenia in all patients. The detection of gram stain and bacterial and fungal cultures were negative for all CSF samples, and none of the CSF samples were contaminated with blood.

**Table 1:** Clinical features and laboratory findings for the 5 patients with neurological manifestations.

					Serum analysis			CSF analysis			
Patient ID	Age (yr) /sex	Clinical classifi-cation*	Date of sample collection (mo-yr)	Clinical features	WBC (x10^3^/mm)	Hematocrit (%)	platelet count (x10^3^/mm)	WBC (x10^3^/mm)	Protein (mg/dl)	Glucose (mg/dl)	CSF/blood sugar ratio (%)
1	24/M	SD	Jun-17	fever, muscle and joint pain, pain in eye socket, nausea, headache, vomiting, rash, irritability, lethargy, photophobia, neck stiffness, alternation of mental status, encephalitis	2.7	35.7	47.8	15	67	124	97
								(80% neutrophils, 20% lymphocytes)			
2	19/F	SD	Aug-17	flushing, gum bleeding, headache, vomiting, free fluid in abdomen/pelvis, abdominal pain, liver enlargement, photophobia, irritability, confusion, loss of consciousness, respiratory failure, generalized fits, encephalitis	2.3	36.7	37.9	119	92.8	-	-
										
3	17/M	SD	Jul-17	fever, muscle and joint pain, pain in eye socket, nausea, headache, vomiting, irritability, neck stiffness, alternation of mental status, encephalitis	5.8	27.8	67.7	246	61.7	97.8	86.5
								(11% neutrophils, 89% lymphocytes)			
4	7/F	SD	Jun-17	fever, muscle and joint pain, pain in eye socket, nausea, headache, vomiting, irritability, alternation of mental status, encephalitis	3.8	29.9	113.9	42	42.1	132.1	78.9
								(83% neutrophils, 17%lymphocytes)			
5	10/F	SD	Nov-17	fever, muscle and joint pain, pain in eye socket, nausea, headache, vomiting, liver enlargement, photophobia, irritability, confusion, alternation of mental status, encephalitis	4.7	36.5	77.9	8	104	78.9	72
								(100% lymphocytes)			

* Clinical classification according to WHO 2009 criteria.

### Virological and serological features of dengue patients with neurological manifestations

DENV-NS1 was detected in the serum of patients 1–4 and CSF of patient 2 (Table 2). Due to insufficient amount of CSF, NS1 Ag test was not performed for the three patients (patients 1, 3, and 4). Quantitative real-time PCR (qRT-PCR) was used to determine the viral genome levels of DENV-2 in the serum and CSF samples from all 5 patients; the highest viral genome levels were detected in the CSF and serum samples of the fatal case-patient (patient 2). DENV-2 isolates were identified in the serum and CSF samples of patients 1 and 2, and the serum sample of patient 3. For patients 4 and 5, virus isolation from their clinical samples failed (Table 2). DENV-2 was detected by conventional PCR in the brain tissue obtained at autopsy on patient 2, the patient who succumbed to the illness. The presence of DENV IgM was detected in the serum and CSF samples of the 4 patients, with the exception of patient 2. Out of the five patients, only one patient (patient 2) had primary infection, whereas all the other 4 patients had secondary infection (Table 2).

**Table 2:** Summary of serological and molecular diagnostic results for the 5 patients with neurological manifestations.

Patient ID	Sample source	No. of days of illness	NS1 Antigen rapid test	Viremia level by real time RT-PCR (log_10_copies/ml)	Virus isolation	DENV conventional RT-PCR	NGS analysis	IgM ratio^a^	IgG titer^b^	Type of infection	Remark
1	252-S^c^	3	+	5.7	+	DENV-2	DENV-2	11.6	43,002	Sec	recovery
	252-C^d^	5	ND	7.8	+	DENV-2	DENV-2	3.2	3,666		
2	257-S	5	+	8.9	+	DENV-2	DENV-2	0.5	294	Pri	death
	257-C	5	+	8.3	+	DENV-2	DENV-2	0.5	95		
	Brain tissue (autopsy)	7				DENV-2					
3	256-S	5	+	6.7	+	DENV-2	DENV-2	9.5	44,876	Sec	recovery
	256-C	5	ND	3.4	-	-	-	12.1	57,677		
4	251-S	3	+	4.2	-	-	-	11.5	72,099	Sec	recovery
	251-C	3	ND	6.3	-	-	-	4.5	8,760		
5	1354-S	10	-	3.6	-	-	-	11.8	60,405	Sec	recovery
	1354-C	10	-	3.9	-	-	-	2.2	16,183		

ND: not done. Pri: primary infection. Sec: secondary infection.

^a^ DENV IgM P/N ratio ≥ 2 considered as positive.

^b^ DENV IgG titer ≥ 29,000 considered as secondary and < 29,000 as primary infection.

^c^S: serum

^d^C:CSF.

### Phylogenetic analysis

**Fig 1:**
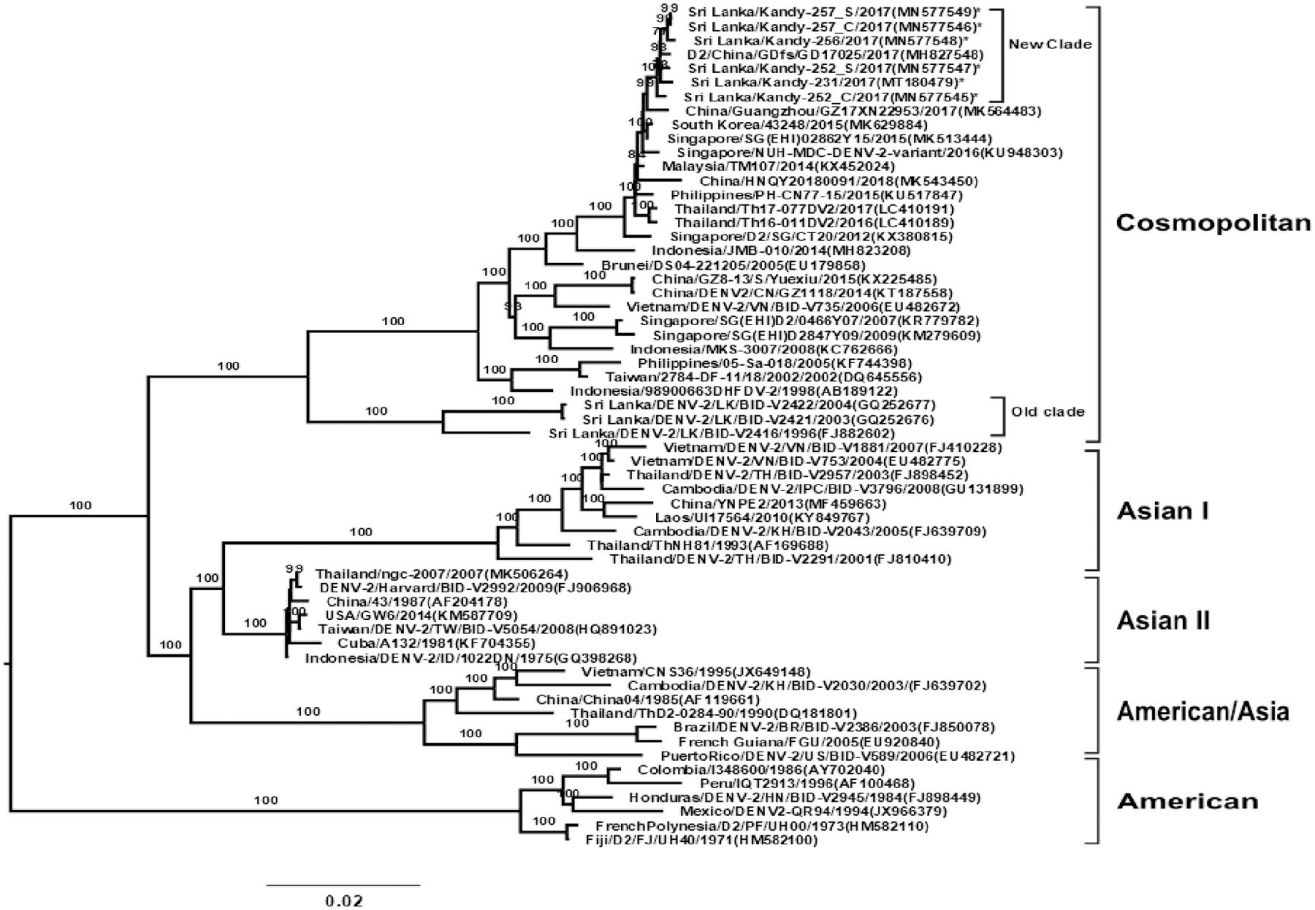
Phylogenetic tree of DENV-2 based on the whole genome. It shows the relationship of the 54 virus strains from different sources, the MT 180479 strain from Sri Lanka, and the 5 isolates (*) of DENV-2 in this study. The 5 isolates in this study and the MT 180479 formed a new clade whereas the strains previously circulating in Sri Lanka belonged to an old clade. The representative strains of each genotype obtained from GenBank are named by country-origin, strain name, year of isolation, and GenBank accession number.

By performing an NGS analysis, the complete genome of DENV-2 was determined from the culture fluids of cells inoculated with the patients’ serum and CSF samples. To compare the genetic background of DENV-2 strains, we compared the sequences of the neurotropic DENV-2 isolates from this study with a closely related reference sequence of DENV-2 (Sri Lanka/Kandy/231-2017/GenBank acc. no. MT180479) which was isolated from serum of non-encephalitis patient who was admitted in the same Kandy hospital during the 2017 dengue outbreak. We determined the genetic relationship of these 5 isolates, the MT 180479 strain and the other 54 DENV-2 strains that have been isolated from geographically diverse areas including Sri Lanka by performing a phylogenetic analysis on their complete genome sequences. The complete genome sequences of the other DENV-strains came from Genbank (Fig 1). The DENV-2 isolates including MT 180479 strain belonged to a new cosmopolitan genotype clade when compared with previously identified DENV-2 isolates (GQ252677, GQ 252676), which circulated in Sri Lanka during 2003 and 2004. The DENV-2 isolates identified in this study were genetically close (99%) to isolates from China, South Korea, Singapore, Malaysia, Thailand, and the Philippines.

### Amino acid variability analysis

To characterize viral amino acid differences by an NGS strategy, the amino acid sequences of the DENV-2 isolates were aligned. The sequence alignment revealed 99% identity among the isolates. Compared with MT180479 strain, amino acid substitutions were identified in 165 sites throughout the complete coding region of the 5 DENV-2 isolates in this study (Table 3). Across the complete genome, the number of positions with variant frequencies > 1% revealed 149 synonymous and 16 nonsynonymous sites among DENV isolates in this study (S1 Table). The variant frequencies > 50% indicated that these variants became predominant in the particular patient (Table 3). The 30 synonymous and 4 nonsynonymous mutations were shared among DENV isolates from the CSF and serum of patients. The fifteen synonymous and one nonsynonymous (K1400R) mutations were found in the virus particles from both serum and CSF of patient 1. The thirty synonymous and three nonsynonymous (S643N, K1400R, V1779I) were shared by the virus particles from serum and CSF of patient 2.

**Table 3:** Synonymous and nonsynonymous variant (>1%) alleles shared among the DENV-2 isolates in this study.

Sample ID^a^	Feature	Nucleo tide position	Amino acid position	Ref: Allele^b^	ALT Allele^c^	Frequency (%)	Change^d^	Sample ID^a^	Feature	Nucleo tide position	Amino acid position	Ref: Allele^b^	ALT Allele^c^	Frequency (%)	Change^d^
252-C	C	276	60	A	G	98.4	Syn-P	257-C	NS3	4647	1517	A	G	99.4	Syn-K
252-S	C	276	60	A	G	100.0	Syn-P	257-S	NS3	4647	1517	A	G	98.4	Syn-K
257-C	C	276	60	A	G	98.4	Syn-P	256-S	NS3	4647	1517	A	G	100.0	Syn-K
257-S	C	276	60	A	G	98.5	Syn-P								
256-S	C	276	60	A	G	100.0	Syn-P	252-C	NS3	5182	1696	T	C	99.0	Syn-L
								252-S	NS3	5182	1696	T	C	98.3	Syn-L
257-C	C	370	92	C	T	99.1	Syn-L	257-C	NS3	5182	1696	T	C	96.0	Syn-L
257-S	C	370	92	C	T	100.0	Syn-L	257-S	NS3	5182	1696	T	C	98.8	Syn-L
								256-S	NS3	5182	1696	T	C	100.0	Syn-L
252-C	pr	678	194	T	C	98.8	Syn-C								
252-S	pr	678	194	T	C	100.0	Syn-C	252-C	NS3	5406	1770	A	G	95.7	Syn-A
257-C	pr	678	194	T	C	99.4	Syn-C	252-S	NS3	5406	1770	A	G	100.0	Syn-A
257-S	pr	678	194	T	C	99.4	Syn-C	257-C	NS3	5406	1770	A	G	98.7	Syn-A
256-S	pr	678	194	T	C	97.7	Syn-C	257-S	NS3	5406	1770	A	G	99.3	Syn-A
								256-S	NS3	5406	1770	A	G	100.0	Syn-A
257-C	M	879	261	A	G	100.0	Syn-T								
257-S	M	879	261	A	G	100.0	Syn-T	257-C	NS3	5431	1779	G	A	100.0	V-I
256-S	M	879	261	A	G	100.0	Syn-T	257-S	NS3	5431	1779	G	A	98.9	V-I
257-C	E	1785	563	T	C	98.3	Syn-L	252-C	NS3	5523	1809	C	T	98.9	Syn-D
257-S	E	1785	563	T	C	98.4	Syn-L	252-S	NS3	5523	1809	C	T	100.0	Syn-D
256-S	E	1785	563	T	C	100.0	Syn-L	257-C	NS3	5523	1809	C	T	100.0	Syn-D
								257-S	NS3	5523	1809	C	T	99.1	Syn-D
257-C	E	2024	643	G	A	99.4	S-N	256-S	NS3	5523	1809	C	T	100.0	Syn-D
257-S	E	2024	643	G	A	98.0	S-N								
								257-C	NS3	5676	1860	C	T	100.0	Syn-L
257-C	E	2095	667	C	T	100.0	Syn-L	257-S	NS3	5676	1860	C	T	98.8	Syn-L
257-S	E	2095	667	C	T	100.0	Syn-L	256-S	NS3	5676	1860	C	T	100.0	Syn-L
256-S	E	2095	667	C	T	96.6	Syn-L								
								252-C	NS3	6174	2026	C	A	100.0	Syn-A
257-C	E	2388	764	C	A	97.2	Syn-V	252-S	NS3	6174	2026	C	A	100.0	Syn-A
257-S	E	2388	764	C	A	96.0	Syn-V	257-C	NS3	6174	2026	C	A	100.0	Syn-A
								257-S	NS3	6174	2026	C	A	100.0	Syn-A
252-C	NS1	2901	935	C	T	99.2	Syn-F	256-S	NS3	6174	2026	C	A	100.0	Syn-A
252-S	NS1	2901	935	C	T	97.8	Syn-F								
257-C	NS1	2901	935	C	T	97.4	Syn-F	252-C	NS4A	6510	2138	C	T	99.0	Syn-L
257-S	NS1	2901	935	C	T	97.0	Syn-F	252-S	NS4A	6510	2138	C	T	100.0	Syn-L
256-S	NS1	2901	935	C	T	100.0	Syn-F	257-C	NS4A	6510	2138	C	T	100.0	Syn-L
								257-S	NS4A	6510	2138	C	T	100.0	Syn-L
257-C	NS1	3327	1077	T	C	100.0	Syn-T	256-S	NS4A	6510	2138	C	T	100.0	Syn-L
257-S	NS1	3327	1077	T	C	100.0	Syn-T								
256-S	NS1	3327	1077	T	C	100.0	Syn-T	252-S	NS4A	6603	2169	A	G	100.0	Syn-K
								257-C	NS4A	6603	2169	A	G	99.4	Syn-K
252-C	NS1	3459	1121	T	C	100.0	Syn-V	257-S	NS4A	6603	2169	A	G	99.0	Syn-K
257-C	NS1	3459	1121	T	C	98.7	Syn-V	256-S	NS4A	6603	2169	A	G	100.0	Syn-K
257-S	NS1	3459	1121	T	C	99.1	Syn-V								
256-S	NS1	3459	1121	T	C	88.9	Syn-V	257-C	2K^e^	6801	2235	T	C	100.0	Syn-L
								257-S	2K	6801	2235	T	C	99.1	Syn-L
252-C	NS2A	3534	1146	A	G	100.0	Syn-L	256-S	2K	6801	2235	T	C	97.0	Syn-L
252-S	NS2A	3534	1146	A	G	100.0	Syn-L								
257-C	NS2A	3534	1146	A	G	99.4	Syn-L	252-C	NS4B	6900	2268	T	C	100.0	Syn-S
257-S	NS2A	3534	1146	A	G	100.0	Syn-L	252-S	NS4B	6900	2268	T	C	100.0	Syn-S
256-S	NS2A	3534	1146	A	G	100.0	Syn-L	257-C	NS4B	6900	2268	T	C	100.0	Syn-S
								257-S	NS4B	6900	2268	T	C	96.1	Syn-S
252-C	NS2A	3597	1167	T	C	99.4	Syn-F	256-S	NS4B	6900	2268	T	C	100.0	Syn-S
252-S	NS2A	3597	1167	T	C	100.0	Syn-F								
257-C	NS2A	3597	1167	T	C	100.0	Syn-F	252-C	NS4B	8028	2644	T	C	97.6	Syn-N
257-S	NS2A	3597	1167	T	C	99.4	Syn-F	252-S	NS4B	8028	2644	T	C	96.8	Syn-N
256-S	NS2A	3597	1167	T	C	100.0	Syn-F	257-C	NS4B	8028	2644	T	C	96.8	Syn-N
								257-S	NS4B	8028	2644	T	C	100.0	Syn-N
252-C	NS2A	3624	1176	T	C	93.9	Syn-S	256-S	NS4B	8028	2644	T	C	100.0	Syn-N
252-S	NS2A	3624	1176	T	C	100.0	Syn-S								
257-C	NS2A	3624	1176	T	C	92.1	Syn-S	257-C	NS4B	8325	2743	C	T	20.0	Syn-Y
257-S	NS2A	3624	1176	T	C	96.5	Syn-S	257-S	NS4B	8325	2743	C	T	23.1	Syn-Y
256-S	NS2A	3624	1176	T	C	100.0	Syn-S								
								257-C	NS4B	8946	2950	G	A	99.2	Syn-E
252-C	NS2B	4295	1400	A	G	100.0	K-R	257-S	NS4B	8946	2950	G	A	66.2	Syn-E
252-S	NS2B	4295	1400	A	G	100.0	K-R	256-S	NS4B	8946	2950	G	A	58.3	Syn-E
257-C	NS2B	4295	1400	A	G	98.5	K-R								
257-S	NS2B	4295	1400	A	G	100.0	K-R	252-C	NS4B	9189	3031	G	A	100.0	Syn-T
256-S	NS2B	4295	1400	A	G	100.0	K-R	252-S	NS4B	9189	3031	G	A	99.4	Syn-T
								257-C	NS4B	9189	3031	G	A	99.4	Syn-T
252-C	NS2B	4488	1464	C	T	100.0	Syn-A	257-S	NS4B	9189	3031	G	A	100.0	Syn-T
252-S	NS2B	4488	1464	C	T	100.0	Syn-A	256-S	NS4B	9189	3031	G	A	100.0	Syn-T
257-C	NS2B	4488	1464	C	T	99.2	Syn-A								
257-S	NS2B	4488	1464	C	T	100.0	Syn-A	257-C	NS4B	9399	3101	T	C	99.4	Syn-N
256-S	NS2B	4488	1464	C	T	100.0	Syn-A	257-S	NS4B	9399	3101	T	C	100.0	Syn-N
								256-S	NS4B	9399	3101	T	C	100.0	Syn-N
257-C	NS3	4587	1497	C	T	99.2	Syn-A								
257-S	NS3	4587	1497	C	T	99.1	Syn-A	252-C	NS5	10606	3504	C	G	7.9	R-G
256-S	NS3	4587	1497	C	T	100.0	Syn-A	256-S	NS5	10606	3504	C	G	31.9	R-G

^a^Same sample ID number means same patient, C: CSF, S: serum.

^b^DENV-2 (Sri Lanka/Kandy/231-2017/MT180479) isolated from serum of non-encephalitis patient during 2017 dengue outbreak was used as reference strain.

^c^Alternative allele in DENV-2 isolates.

^d^Synonymous and nonsynonymous mutations.

^e^2K peptide, 17 amino acid peptide linking NS4A with NS4B of DENV.

**Fig 2:**
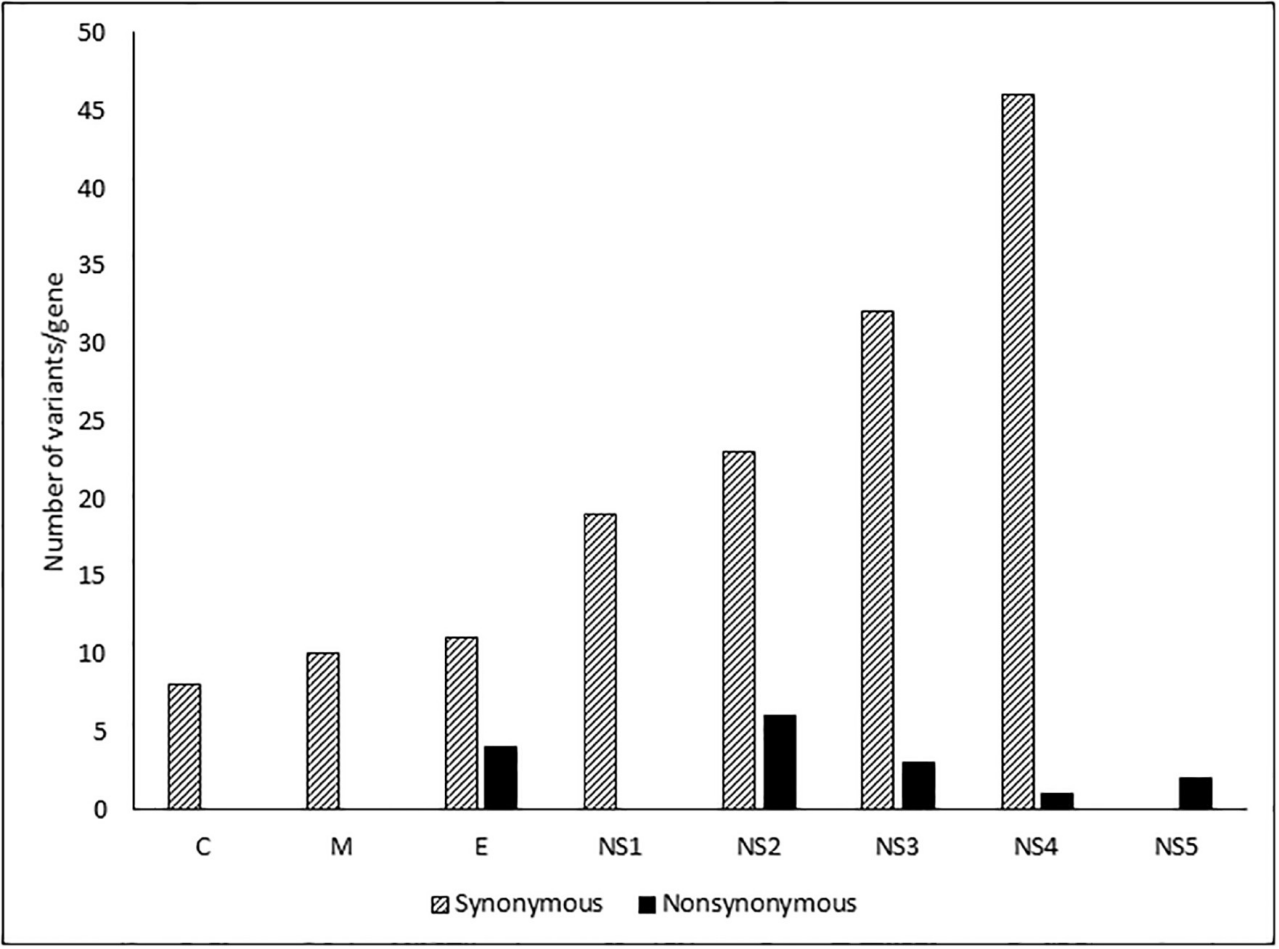
Number of positions with variant incidence > 1% per gene among the DENV-2 isolates.

We analyzed the total number of synonymous and nonsynonymous mutations in each gene, independent of the frequency (Fig 2). The number of synonymous variants was higher than the number of nonsynonymous substitutions at each site. The greater variability was observed among the nonstructural genes than the structural genes. There was significant association between the mutations with DENV genes (p = <0.0001, chi-square test). Significantly higher proportion of nonsynonymous mutations was observed in the NS2 gene compared to other genes. There was no difference in the ratios of frequencies of non-synonymous to synonymous mutations (nS/S) between the structural and non-structural genes (p = 0.85, Mann Whitney U test) in (S1 Fig).

## Discussion

The 2017 dengue outbreak in Sri Lanka was the largest in the island’s history [13]. Out of 295 dengue-suspected patients, 5 patients (1.7%) who presented with neurological symptoms and confirmed to have dengue encephalitis were the subjects of this study. DENV infections associated with neurological manifestations have been reported in Thailand, India, Vietnam, and Cambodia at 1–20% [32–36], in Brazil at 21–47% [37, 38], and in Puerto Rico at 26% of patients [39]. Neurological manifestations caused by DENV have been reported in patients with ages ranging from 3 months to 60 years [6]. In the present study, 2 children and 3 adults were identified with DENV-associated neurological manifestations. CNS involvement occurs 4–30 days after the first dengue symptoms appear, with a median of 12 days after the onset of fever, and CNS symptoms were identified between days 3–10 after the onset of fever in our study [38].

The protein NS1 is present at high concentrations in the sera from dengue-infected patients during the early clinical phase of the disease and can be found between days 1–9 after the onset of fever in samples from primary or secondary dengue-infected patients [40]. The detection of NS1 in serum samples was positive in 4 of the 5 patients in this study, starting between 3–5 days after the onset of illness. The patient who was negative for dengue NS1 Ag was tested for the presence of this antigen on day 10 of the illness The detection of dengue IgM in CSF has been shown to have a high specificity (97%) for the diagnosis of neurologic dengue and might be associated with the neurovirulence of DENV and its ability to cause encephalitis [41]. Together, our results showed that all patients were DENV-IgM-positive, except for the fatal case. In a previous study, dengue RNA was found in the CSF from 7 out of 13 Brazilian patients, and the CSF viral load was lower than 1,000 copies/ml [37]. However in the present study, dengue viral genome detected in CSF samples from 3 patients showed high viral loads (> 1,000,000 copies/ml) and the remaining 2 patients had viral loads higher than 1,000 copies/ml.

A number of studies have reported the presence of DENV RNA and the isolation of DENV from CSF samples taken from encephalitis patients. However, only a few studies, including one study that reported the complete genome sequence for DENV-4, two studies that reported partial C-PrM-E sequences for DENV-3, one study that reported the complete genome sequence for DENV-3 and two studies that reported partial E-NS1 sequences for DENV-2, have examined the genetic characteristics of neurotropic DENV. All of these DENV were isolated from CSF samples [8, 42–45]. To our knowledge, this is the first report to describe the complete genome and the molecular and virological characteristics of DENV-2 isolated from CSF samples from fatal and non-fatal dengue case-patients with neurological manifestations. Compared with other dengue serotypes, DENV-2 and DENV-3 appear to be more frequently associated with encephalitis and other neurological manifestations, regardless of whether it is primary or secondary infections [43, 45]. In a meta-analysis examining the severity of dengue during primary or secondary infection with different DENV serotypes, secondary infections with DENV-2 were reported to be associated with severe cases [46]. In our study, 4 out of 5 patients with SD was due to secondary infections. However, only one case was associated with a primary DENV-2 infection and this might be due to other viral and/ or host factors. The detection of DENV in the brain and CSF by PCR and virus isolation and the detection of NS1 and dengue IgM provided strong evidences that DENV has neurovirulent properties. The evidence of DENV in the CSF, as assessed by RT-PCR and ELISA (NS1/IgM) in this study, is consistent with a CNS infection.

A complete genomic phylogenetic analysis revealed that DENV-2 isolates from the serum and CSF of the same patients showed high similarity and appeared in the same branch. The full genomes of serum- and CSF-derived DENV-2 from the same patient shared 99.7% similarity, indicating the virus dissemination across the blood-brain barrier. Two novel mutations, S643N (E) and V1779I (NS3), which have not been reported previously, were identified in CSF and serum of patient 2. Furthermore, our study compared the genetic background of DENV-2 strains from the 5 dengue encephalitis patients in this study and of DENV-2 strain (MT180479) from non-encephalitis patient who was admitted to the hospital where the 5 patients were also admitted during the dengue outbreak. There were 16 amino acid changes found in neurotropic DENV-2 isolates compared with MT180479 strain. However, whether these amino acid changes are associated with the neurovirulence properties of these DENV-2 isolates remains unclear. Both viral mutations and the genetic or immunological status of the hosts may contribute to the development of dengue encephalitis in patients [47–49]. In addition, further studies including cell tropism studies using in vivo and in vitro experiment are necessary to elucidate the virulence of these isolates.

In conclusion, our study reported for the first time the isolation and the complete genome analysis of a new cosmopolitan genotype clade of neurotropic DENV-2 isolated from serum and CSF samples taken from the same patients during the unprecedented dengue outbreak in Sri Lanka, in 2017. Our findings suggested that pathogenic DENV-2 caused neurological complications, including one fatal case. We concluded that genetic variations were increased among the non-structural DENV-2 genes. In addition, dengue should be suspected in patients with neurological manifestations in dengue-endemic areas, regardless of whether the patient presents with classical dengue features.

## Supporting information

S1 Table Synonymous and nonsynonymous variant (>1%) alleles among the DENV-2 isolates in this study. (DOCX)

S1 Fig The ratios of frequencies of nonsynonymous and synonymous mutations (nS/S) among strctural and non-structural genes. (TIF)
