# Supplementary material for: Complete genome analysis and characterization of neurotropic dengue virus 2 cosmopolitan genotype isolated from the cerebrospinal fluid of encephalitis patients
Source: PLoS One. 2020 Jun 18;15(6):e0234508. doi: 10.1371/journal.pone.0234508 (PMC7302667; doi:10.1371/journal.pone.0234508)
Supplement: S1 Table — (DOCX) [file pone.0234508.s001.docx]

**S1 Table. Synonymous and nonsynonymous variant (>1%) alleles among DENV-2 isolates in this study.**

|  |  |  |  |  |  |  |  |  |  |  |  |  |  |  |  |
| --- | --- | --- | --- | --- | --- | --- | --- | --- | --- | --- | --- | --- | --- | --- | --- |
|  |  |  |  | Amino acid | |  | Reference^b^ | | |  | ALT^c^ | | |  |  |
| Patient | Sample ID^a^ | Feature | Nucleotide position | Whole gene position | Specific gene position |  | Allele | Codon | Amino acid |  | Allele | Codon | Amino acid | Frequency | Change^d^ |
| 1 | 252-C | C | 276 | 60 | 60 |  | A | CCA | P |  | G | CCG | P | 98.44% | Synonymous |
|  | 252-C | pr | 450 | 118 | 4 |  | C | ACC | T |  | T | ACT | T | 12.04% | Synonymous |
|  | 252-C | pr | 477 | 127 | 13 |  | C | ATC | I |  | T | ATT | I | 98.96% | Synonymous |
|  | 252-C | pr | 678 | 194 | 80 |  | T | TGT | C |  | C | TGC | C | 98.77% | Synonymous |
|  | 252-C | E | 1152 | 352 | 72 |  | T | TCT | S |  | G | TCG | S | 98.87% | Synonymous |
|  | 252-C | NS1 | 2709 | 871 | 96 |  | C | ATC | I |  | T | ATT | I | 99.10% | Synonymous |
|  | 252-C | NS1 | 2829 | 911 | 136 |  | T | GAT | D |  | C | GAC | D | 96.02% | Synonymous |
|  | 252-C | NS1 | 2901 | 935 | 160 |  | C | TTC | F |  | T | TTT | F | 99.22% | Synonymous |
|  | 252-C | NS1 | 3030 | 978 | 203 |  | A | GAA | E |  | G | GAG | E | 100% | Synonymous |
|  | 252-C | NS1 | 3075 | 993 | 218 |  | T | ATT | I |  | C | ATC | I | 99.22% | Synonymous |
|  | 252-C | NS1 | 3459 | 1121 | 346 |  | T | GTT | V |  | C | GTC | V | 100% | Synonymous |
|  | 252-C | NS1 | 3462 | 1122 | 347 |  | C | AAC | N |  | T | AAT | N | 64.10% | Synonymous |
|  | 252-C | NS2A | 3481 | 1129 | 2 |  | T | TAT | Y |  | C | CAT | H | 100% | Nonsynonymous |
|  | 252-C | NS2A | 3534 | 1146 | 19 |  | A | CTA | L |  | G | CTG | L | 100% | Synonymous |
|  | 252-C | NS2A | 3597 | 1167 | 40 |  | T | TTT | F |  | C | TTC | F | 99.42% | Synonymous |
|  | 252-C | NS2A | 3624 | 1176 | 49 |  | T | TCT | S |  | C | TCC | S | 93.87% | Synonymous |
|  | 252-C | NS2A | 3702 | 1202 | 75 |  | T | CTT | L |  | A | CTA | L | 97.50% | Synonymous |
|  | 252-C | NS2B | 4295 | 1400 | 55 |  | A | AAA | K |  | G | AGA | R | 100% | Nonsynonymous |
|  | 252-C | NS2B | 4488 | 1464 | 119 |  | C | GCC | A |  | T | GCT | A | 100% | Synonymous |
|  | 252-C | NS3 | 4767 | 1557 | 82 |  | T | GGT | G |  | A | GGA | G | 100% | Synonymous |
|  | 252-C | NS3 | 5182 | 1696 | 221 |  | T | TTG | L |  | C | CTG | L | 99.02% | Synonymous |
|  | 252-C | NS3 | 5406 | 1770 | 295 |  | A | GCA | A |  | G | GCG | A | 95.68% | Synonymous |
|  | 252-C | NS3 | 5523 | 1809 | 334 |  | C | GAC | D |  | T | GAT | D | 98.87% | Synonymous |
|  | 252-C | NS3 | 6174 | 2026 | 551 |  | C | GCC | A |  | A | GCA | A | 100% | Synonymous |
|  | 252-C | NS4A | 6471 | 2125 | 32 |  | T | CAT | H |  | C | CAC | H | 100% | Synonymous |
|  | 252-C | NS4A | 6510 | 2138 | 45 |  | C | CTC | L |  | T | CTT | L | 99.00% | Synonymous |
|  | 252-C | NS4B | 6900 | 2268 | 25 |  | T | AGT | S |  | C | AGC | S | 100% | Synonymous |
|  | 252-C | NS4B | 8028 | 2644 | 401 |  | T | AAT | N |  | C | AAC | N | 95.76% | Synonymous |
|  | 252-C | NS4B | 8106 | 2670 | 427 |  | C | TGC | C |  | T | TGT | C | 6.50% | Synonymous |
|  | 252-C | NS4B | 8199 | 2701 | 458 |  | C | CTC | L |  | T | CTT | L | 100% | Synonymous |
|  | 252-C | NS4B | 8286 | 2730 | 487 |  | A | TTA | L |  | G | TTG | L | 100% | Synonymous |
|  | 252-C | NS4B | 8319 | 2741 | 498 |  | C | GCC | A |  | T | GCT | A | 100% | Synonymous |
|  | 252-C | NS4B | 8574 | 2826 | 583 |  | T | ATT | I |  | C | ATC | I | 90.54% | Synonymous |
|  | 252-C | NS4B | 9189 | 3031 | 788 |  | G | ACG | T |  | A | ACA | T | 100% | Synonymous |
|  | 252-C | NS4B | 9462 | 3122 | 879 |  | C | AGC | S |  | T | AGT | S | 100% | Synonymous |
|  | 252-C | NS5 | 10606 | 3504 | 113 |  | C | CGA | R |  | G | GGA | G | 7.87% | Nonsynonymous |
|  | 252-S | C | 219 | 41 | 41 |  | A | CGA | R |  | G | CGG | R | 93.55% | Synonymous |
|  | 252-S | C | 276 | 60 | 60 |  | A | CCA | P |  | G | CCG | P | 100% | Synonymous |
|  | 252-S | pr | 678 | 194 | 80 |  | T | TGT | C |  | C | TGC | C | 100% | Synonymous |
|  | 252-S | E | 1410 | 438 | 158 |  | C | CAC | H |  | T | CAT | H | 100% | Synonymous |
|  | 252-S | E | 1555 | 487 | 207 |  | C | CTG | L |  | T | TTG | L | 93.33% | Synonymous |
|  | 252-S | E | 1928 | 611 | 331 |  | C | TCT | S |  | T | TTT | F | 98.70% | Nonsynonymous |
|  | 252-S | NS1 | 2901 | 935 | 160 |  | C | TTC | F |  | T | TTT | F | 97.83% | Synonymous |
|  | 252-S | NS1 | 3201 | 1035 | 260 |  | T | TAT | Y |  | C | TAC | Y | 98.81% | Synonymous |
|  | 252-S | NS1 | 3276 | 1060 | 285 |  | G | GTG | V |  | A | GTA | V | 100% | Synonymous |
|  | 252-S | NS2A | 3534 | 1146 | 19 |  | A | CTA | L |  | G | CTG | L | 100% | Synonymous |
|  | 252-S | NS2A | 3597 | 1167 | 40 |  | T | TTT | F |  | C | TTC | F | 100% | Synonymous |
|  | 252-S | NS2A | 3624 | 1176 | 49 |  | T | TCT | S |  | C | TCC | S | 100% | Synonymous |
|  | 252-S | NS2A | 3855 | 1253 | 126 |  | G | GCG | A |  | A | GCA | A | 100% | Synonymous |
|  | 252-S | NS2B | 4295 | 1400 | 55 |  | A | AAA | K |  | G | AGA | R | 100% | Nonsynonymous |
|  | 252-S | NS2B | 4368 | 1424 | 79 |  | G | TCG | S |  | A | TCA | S | 100% | Synonymous |
|  | 252-S | NS2B | 4488 | 1464 | 119 |  | C | GCC | A |  | T | GCT | A | 100% | Synonymous |
|  | 252-S | NS3 | 4635 | 1513 | 38 |  | C | GCC | A |  | T | GCT | A | 100% | Synonymous |
|  | 252-S | NS3 | 5182 | 1696 | 221 |  | T | TTG | L |  | C | CTG | L | 98.28% | Synonymous |
|  | 252-S | NS3 | 5207 | 1704 | 229 |  | C | GCT | A |  | T | GTT | V | 100% | Nonsynonymous |
|  | 252-S | NS3 | 5406 | 1770 | 295 |  | A | GCA | A |  | G | GCG | A | 100% | Synonymous |
|  | 252-S | NS3 | 5523 | 1809 | 334 |  | C | GAC | D |  | T | GAT | D | 100% | Synonymous |
|  | 252-S | NS3 | 6174 | 2026 | 551 |  | C | GCC | A |  | A | GCA | A | 100% | Synonymous |
|  | 252-S | NS4A | 6510 | 2138 | 45 |  | C | CTC | L |  | T | CTT | L | 100% | Synonymous |
|  | 252-S | NS4A | 6603 | 2169 | 76 |  | A | AAA | K |  | G | AAG | K | 100% | Synonymous |
|  | 252-S | NS4A | 6643 | 2183 | 90 |  | A | ACA | T |  | G | GCA | A | 100% | Nonsynonymous |
|  | 252-S | NS4B | 6900 | 2268 | 25 |  | T | AGT | S |  | C | AGC | S | 100% | Synonymous |
|  | 252-S | NS4B | 7635 | 2513 | 270 |  | G | AAG | K |  | A | AAA | K | 81.69% | Synonymous |
|  | 252-S | NS4B | 8028 | 2644 | 401 |  | T | AAT | N |  | C | AAC | N | 96.77% | Synonymous |
|  | 252-S | NS4B | 9189 | 3031 | 788 |  | G | ACG | T |  | A | ACA | T | 98.36% | Synonymous |
| 2 | 257-C | C | 276 | 60 | 60 |  | A | CCA | P |  | G | CCG | P | 98.40% | Synonymous |
|  | 257-C | C | 370 | 92 | 92 |  | C | CTG | L |  | T | TTG | L | 99.08% | Synonymous |
|  | 257-C | pr | 678 | 194 | 80 |  | T | TGT | C |  | C | TGC | C | 99.42% | Synonymous |
|  | 257-C | M | 879 | 261 | 56 |  | A | ACA | T |  | G | ACG | T | 100% | Synonymous |
|  | 257-C | E | 1785 | 563 | 283 |  | T | CTT | L |  | C | CTC | L | 98.25% | Synonymous |
|  | 257-C | E | 2024 | 643 | 363 |  | G | AGC | S |  | A | AAC | N | 99.43% | Nonsynonymous |
|  | 257-C | E | 2095 | 667 | 387 |  | C | CTG | L |  | T | TTG | L | 100% | Synonymous |
|  | 257-C | E | 2388 | 764 | 484 |  | C | GTC | V |  | A | GTA | V | 97.18% | Synonymous |
|  | 257-C | NS1 | 2901 | 935 | 160 |  | C | TTC | F |  | T | TTT | F | 97.40% | Synonymous |
|  | 257-C | NS1 | 3327 | 1077 | 302 |  | T | ACT | T |  | C | ACC | T | 100% | Synonymous |
|  | 257-C | NS1 | 3459 | 1121 | 346 |  | T | GTT | V |  | C | GTC | V | 98.70% | Synonymous |
|  | 257-C | NS2A | 3534 | 1146 | 19 |  | A | CTA | L |  | G | CTG | L | 99.35% | Synonymous |
|  | 257-C | NS2A | 3597 | 1167 | 40 |  | T | TTT | F |  | C | TTC | F | 100% | Synonymous |
|  | 257-C | NS2A | 3624 | 1176 | 49 |  | T | TCT | S |  | C | TCC | S | 92.05% | Synonymous |
|  | 257-C | NS2B | 4295 | 1400 | 55 |  | A | AAA | K |  | G | AGA | R | 98.46% | Nonsynonymous |
|  | 257-C | NS2B | 4488 | 1464 | 119 |  | C | GCC | A |  | T | GCT | A | 99.21% | Synonymous |
|  | 257-C | NS3 | 4587 | 1497 | 22 |  | C | GCC | A |  | T | GCT | A | 99.24% | Synonymous |
|  | 257-C | NS3 | 4647 | 1517 | 42 |  | A | AAA | K |  | G | AAG | K | 99.38% | Synonymous |
|  | 257-C | NS3 | 5182 | 1696 | 221 |  | T | TTG | L |  | C | CTG | L | 96% | Synonymous |
|  | 257-C | NS3 | 5406 | 1770 | 295 |  | A | GCA | A |  | G | GCG | A | 98.74% | Synonymous |
|  | 257-C | NS3 | 5431 | 1779 | 304 |  | G | GTA | V |  | A | ATA | I | 100% | Nonsynonymous |
|  | 257-C | NS3 | 5523 | 1809 | 334 |  | C | GAC | D |  | T | GAT | D | 100% | Synonymous |
|  | 257-C | NS3 | 5676 | 1860 | 385 |  | C | CTC | L |  | T | CTT | L | 100% | Synonymous |
|  | 257-C | NS3 | 6174 | 2026 | 551 |  | C | GCC | A |  | A | GCA | A | 100% | Synonymous |
|  | 257-C | NS4A | 6510 | 2138 | 45 |  | C | CTC | L |  | T | CTT | L | 100% | Synonymous |
|  | 257-C | NS4A | 6603 | 2169 | 76 |  | A | AAA | K |  | G | AAG | K | 99.37% | Synonymous |
|  | 257-C | 2K^e^ | 6801 | 2235 | 15 |  | T | CTT | L |  | C | CTC | L | 100% | Synonymous |
|  | 257-C | NS4B | 6900 | 2268 | 25 |  | T | AGT | S |  | C | AGC | S | 100% | Synonymous |
|  | 257-C | NS4B | 8028 | 2644 | 401 |  | T | AAT | N |  | C | AAC | N | 96.84% | Synonymous |
|  | 257-C | NS4B | 8325 | 2743 | 500 |  | C | TAC | Y |  | T | TAT | Y | 20% | Synonymous |
|  | 257-C | NS4B | 8946 | 2950 | 707 |  | G | GAG | E |  | A | GAA | E | 99.19% | Synonymous |
|  | 257-C | NS4B | 9189 | 3031 | 788 |  | G | ACG | T |  | A | ACA | T | 99.41% | Synonymous |
|  | 257-C | NS4B | 9399 | 3101 | 858 |  | T | AAT | N |  | C | AAC | N | 99.40% | Synonymous |
|  | 257-S | C | 276 | 60 | 60 |  | A | CCA | P |  | G | CCG | P | 98.53% | Synonymous |
|  | 257-S | C | 370 | 92 | 92 |  | C | CTG | L |  | T | TTG | L | 100% | Synonymous |
|  | 257-S | pr | 678 | 194 | 80 |  | T | TGT | C |  | C | TGC | C | 99.38% | Synonymous |
|  | 257-S | M | 879 | 261 | 56 |  | A | ACA | T |  | G | ACG | T | 100% | Synonymous |
|  | 257-S | E | 1546 | 484 | 204 |  | A | AAA | K |  | C | CAA | Q | 21.84% | Nonsynonymous |
|  | 257-S | E | 1785 | 563 | 283 |  | T | CTT | L |  | C | CTC | L | 98.39% | Synonymous |
|  | 257-S | E | 2024 | 643 | 363 |  | G | AGC | S |  | A | AAC | N | 98.04% | Nonsynonymous |
|  | 257-S | E | 2095 | 667 | 387 |  | C | CTG | L |  | T | TTG | L | 100% | Synonymous |
|  | 257-S | E | 2388 | 764 | 484 |  | C | GTC | V |  | A | GTA | V | 96% | Synonymous |
|  | 257-S | NS1 | 2901 | 935 | 160 |  | C | TTC | F |  | T | TTT | F | 97% | Synonymous |
|  | 257-S | NS1 | 3327 | 1077 | 302 |  | T | ACT | T |  | C | ACC | T | 100% | Synonymous |
|  | 257-S | NS1 | 3459 | 1121 | 346 |  | T | GTT | V |  | C | GTC | V | 99.09% | Synonymous |
|  | 257-S | NS2A | 3534 | 1146 | 19 |  | A | CTA | L |  | G | CTG | L | 100% | Synonymous |
|  | 257-S | NS2A | 3597 | 1167 | 40 |  | T | TTT | F |  | C | TTC | F | 99.37% | Synonymous |
|  | 257-S | NS2A | 3624 | 1176 | 49 |  | T | TCT | S |  | C | TCC | S | 96.50% | Synonymous |
|  | 257-S | NS2B | 4295 | 1400 | 55 |  | A | AAA | K |  | G | AGA | R | 100% | Nonsynonymous |
|  | 257-S | NS2B | 4488 | 1464 | 119 |  | C | GCC | A |  | T | GCT | A | 100% | Synonymous |
|  | 257-S | NS3 | 4587 | 1497 | 22 |  | C | GCC | A |  | T | GCT | A | 99.07% | Synonymous |
|  | 257-S | NS3 | 4647 | 1517 | 42 |  | A | AAA | K |  | G | AAG | K | 98.41% | Synonymous |
|  | 257-S | NS3 | 5182 | 1696 | 221 |  | T | TTG | L |  | C | CTG | L | 98.82% | Synonymous |
|  | 257-S | NS3 | 5406 | 1770 | 295 |  | A | GCA | A |  | G | GCG | A | 99.26% | Synonymous |
|  | 257-S | NS3 | 5431 | 1779 | 304 |  | G | GTA | V |  | A | ATA | I | 98.86% | Nonsynonymous |
|  | 257-S | NS3 | 5523 | 1809 | 334 |  | C | GAC | D |  | T | GAT | D | 99.08% | Synonymous |
|  | 257-S | NS3 | 5676 | 1860 | 385 |  | C | CTC | L |  | T | CTT | L | 98.78% | Synonymous |
|  | 257-S | NS3 | 6174 | 2026 | 551 |  | C | GCC | A |  | A | GCA | A | 100% | Synonymous |
|  | 257-S | NS4A | 6510 | 2138 | 45 |  | C | CTC | L |  | T | CTT | L | 100% | Synonymous |
|  | 257-S | NS4A | 6603 | 2169 | 76 |  | A | AAA | K |  | G | AAG | K | 99.04% | Synonymous |
|  | 257-S | 2K | 6801 | 2235 | 15 |  | T | CTT | L |  | C | CTC | L | 99.11% | Synonymous |
|  | 257-S | NS4B | 6900 | 2268 | 25 |  | T | AGT | S |  | C | AGC | S | 96.05% | Synonymous |
|  | 257-S | NS4B | 8028 | 2644 | 401 |  | T | AAT | N |  | C | AAC | N | 100% | Synonymous |
|  | 257-S | NS4B | 8325 | 2743 | 500 |  | C | TAC | Y |  | T | TAT | Y | 23.08% | Synonymous |
|  | 257-S | NS4B | 8946 | 2950 | 707 |  | G | GAG | E |  | A | GAA | E | 66.18% | Synonymous |
|  | 257-S | NS4B | 9189 | 3031 | 788 |  | G | ACG | T |  | A | ACA | T | 100% | Synonymous |
|  | 257-S | NS4B | 9399 | 3101 | 858 |  | T | AAT | N |  | C | AAC | N | 100% | Synonymous |
| 3 | 256-S | C | 276 | 60 | 60 |  | A | CCA | P |  | G | CCG | P | 100% | Synonymous |
|  | 256-S | pr | 678 | 194 | 80 |  | T | TGT | C |  | C | TGC | C | 97.73% | Synonymous |
|  | 256-S | M | 879 | 261 | 56 |  | A | ACA | T |  | G | ACG | T | 100% | Synonymous |
|  | 256-S | E | 1785 | 563 | 283 |  | T | CTT | L |  | C | CTC | L | 100% | Synonymous |
|  | 256-S | E | 2095 | 667 | 387 |  | C | CTG | L |  | T | TTG | L | 96.55% | Synonymous |
|  | 256-S | NS1 | 2901 | 935 | 160 |  | C | TTC | F |  | T | TTT | F | 100% | Synonymous |
|  | 256-S | NS1 | 3327 | 1077 | 302 |  | T | ACT | T |  | C | ACC | T | 100% | Synonymous |
|  | 256-S | NS1 | 3459 | 1121 | 346 |  | T | GTT | V |  | C | GTC | V | 88.89% | Synonymous |
|  | 256-S | NS2A | 3534 | 1146 | 19 |  | A | CTA | L |  | G | CTG | L | 100% | Synonymous |
|  | 256-S | NS2A | 3597 | 1167 | 40 |  | T | TTT | F |  | C | TTC | F | 100% | Synonymous |
|  | 256-S | NS2A | 3624 | 1176 | 49 |  | T | TCT | S |  | C | TCC | S | 100% | Synonymous |
|  | 256-S | NS2B | 4295 | 1400 | 55 |  | A | AAA | K |  | G | AGA | R | 100% | Nonsynonymous |
|  | 256-S | NS2B | 4488 | 1464 | 119 |  | C | GCC | A |  | T | GCT | A | 100% | Synonymous |
|  | 256-S | NS3 | 4587 | 1497 | 22 |  | C | GCC | A |  | T | GCT | A | 100% | Synonymous |
|  | 256-S | NS3 | 4647 | 1517 | 42 |  | A | AAA | K |  | G | AAG | K | 100% | Synonymous |
|  | 256-S | NS3 | 4662 | 1522 | 47 |  | C | CAC | H |  | T | CAT | H | 100% | Synonymous |
|  | 256-S | NS3 | 5182 | 1696 | 221 |  | T | TTG | L |  | C | CTG | L | 100% | Synonymous |
|  | 256-S | NS3 | 5406 | 1770 | 295 |  | A | GCA | A |  | G | GCG | A | 100% | Synonymous |
|  | 256-S | NS3 | 5523 | 1809 | 334 |  | C | GAC | D |  | T | GAT | D | 100% | Synonymous |
|  | 256-S | NS3 | 5676 | 1860 | 385 |  | C | CTC | L |  | T | CTT | L | 100% | Synonymous |
|  | 256-S | NS3 | 6174 | 2026 | 551 |  | C | GCC | A |  | A | GCA | A | 100% | Synonymous |
|  | 256-S | NS4A | 6510 | 2138 | 45 |  | C | CTC | L |  | T | CTT | L | 100% | Synonymous |
|  | 256-S | NS4A | 6603 | 2169 | 76 |  | A | AAA | K |  | G | AAG | K | 100% | Synonymous |
|  | 256-S | 2K | 6801 | 2235 | 15 |  | T | CTT | L |  | C | CTC | L | 96.97% | Synonymous |
|  | 256-S | NS4B | 6900 | 2268 | 25 |  | T | AGT | S |  | C | AGC | S | 100% | Synonymous |
|  | 256-S | NS4B | 7092 | 2332 | 89 |  | C | ATC | I |  | T | ATT | I | 100% | Synonymous |
|  | 256-S | NS4B | 7995 | 2633 | 390 |  | A | ACA | T |  | G | ACG | T | 100% | Synonymous |
|  | 256-S | NS4B | 8028 | 2644 | 401 |  | T | AAT | N |  | C | AAC | N | 100% | Synonymous |
|  | 256-S | NS4B | 8946 | 2950 | 707 |  | G | GAG | E |  | A | GAA | E | 58.33% | Synonymous |
|  | 256-S | NS4B | 9066 | 2990 | 747 |  | C | TCC | S |  | T | TCT | S | 96.43% | Synonymous |
|  | 256-S | NS4B | 9189 | 3031 | 788 |  | G | ACG | T |  | A | ACA | T | 100% | Synonymous |
|  | 256-S | NS4B | 9399 | 3101 | 858 |  | T | AAT | N |  | C | AAC | N | 100% | Synonymous |
|  | 256-S | NS5 | 10606 | 3504 | 113 |  | C | CGA | R |  | G | GGA | G | 31.91% | Nonsynonymous |
|  |  |  |  |  |  |  |  |  |  |  |  |  |  |  |  |

a. **Sample ID-C, CSF and –S, serum of same patient.**

b. DENV-2(Sri Lanka/Kandy/231-2017/MT180479) was used as reference strain which isolated from serum in non-neurological manifestation of patient during 2017 dengue outbreak.

c. Alternative allele in DENV-2 isolates.

d. Synonymous and nonsynonymous mutations.

e. 2K peptide, 17 amino acid peptide linking NS4A with NS4B of DENV.
