# Supplementary figures and images for: Complete genome analysis and characterization of neurotropic dengue virus 2 cosmopolitan genotype isolated from the cerebrospinal fluid of encephalitis patients
Source: PLoS One. 2020 Jun 18;15(6):e0234508. doi: 10.1371/journal.pone.0234508 (PMC7302667; doi:10.1371/journal.pone.0234508)

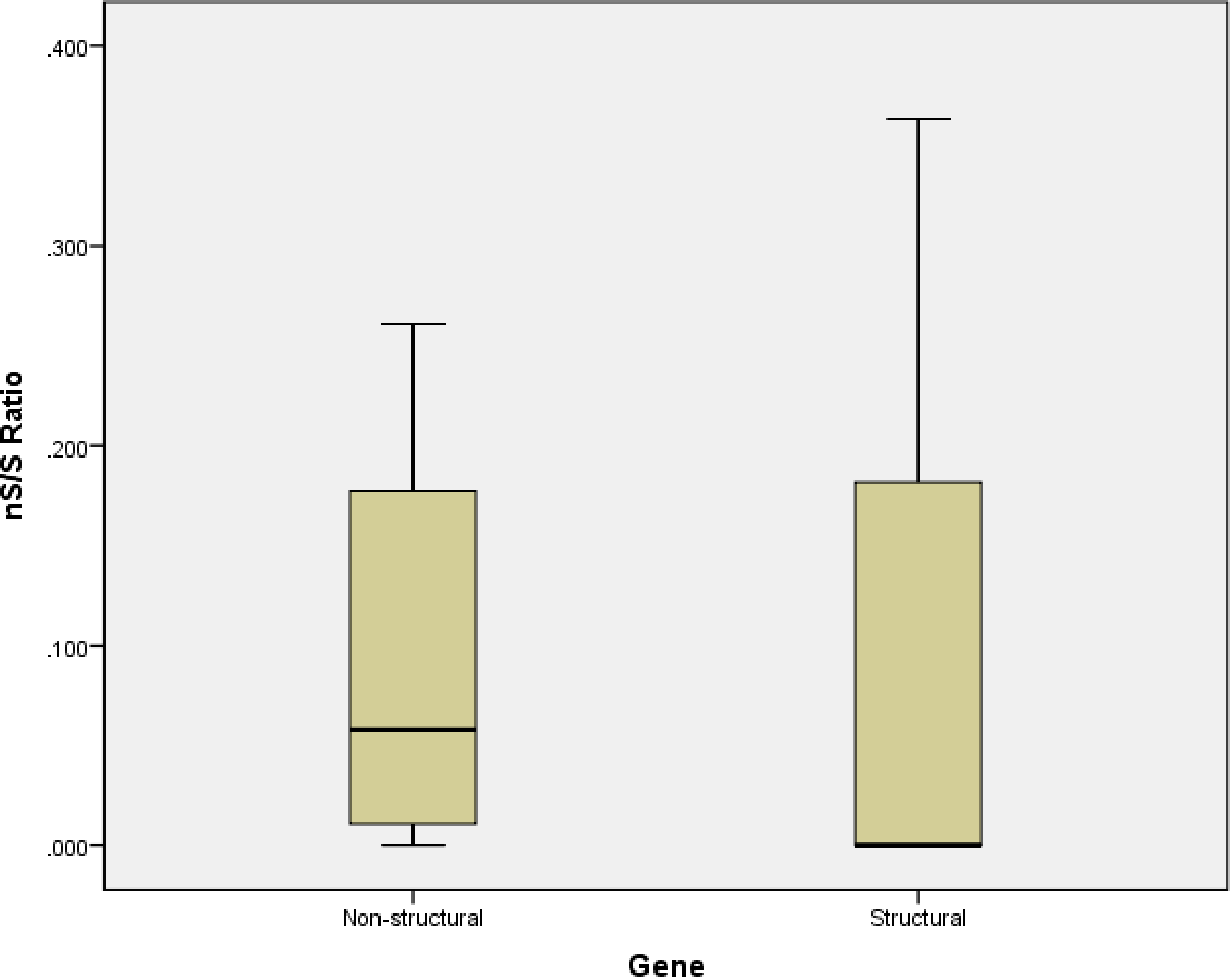

Supplement: S1 Fig — (TIF) [file pone.0234508.s002.tif]
